# Explicit but Not Implicit Memory Predicts Ultimate Attainment in the Native Language

**DOI:** 10.3389/fpsyg.2020.569586

**Published:** 2020-09-25

**Authors:** Miquel Llompart, Ewa Dąbrowska

**Affiliations:** ^1^Chair of Language and Cognition, Department of English and American Studies, Friedrich Alexander University Erlangen-Nuremberg, Erlangen, Germany; ^2^Department of English Language and Linguistics, University of Birmingham, Birmingham, United Kingdom

**Keywords:** implicit memory, explicit memory, ultimate attainment, individual differences, declarative/procedural model, usage-based models

## Abstract

The present paper examines the relationship between explicit and implicit memory and ultimate attainment in the native language. Two groups of native speakers of English with different levels of academic attainment (i.e., high vs. low) took part in three language tasks which assessed grammar, vocabulary and collocational knowledge, as well as phonological short-term memory (assessed using a forward digit-span task), explicit associative memory (assessed using a paired-associates task) and implicit memory (assessed using a deterministic serial reaction time task). Results revealed strong relationships between phonological short-term memory and explicit associative memory on the one hand and the three language tasks on the other hand, and no relation between linguistic performance and implicit memory. Taken together, these results cast doubts on the common assumption that L1 grammar learning depends almost entirely on implicit memory and align with the claims of usage-based models of language acquisition that grammatical and lexical knowledge depend on the same cognitive mechanisms.

## Introduction

Learning a language involves storing vast amounts of information. This means that memory plays an essential role in language learning, knowledge and use (Ullman, 2016). Traditionally, memory has been divided into two well-studied systems when considered in relation to language learning: declarative/explicit memory and procedural/implicit memory^1^. The declarative/explicit memory system, involving the hippocampus and medial temporal lobe structures, is thought to be mainly in charge of the conscious learning, storage and recollection of factual information (Henke, 2010; Squire and Wixted, 2011; Ullman, 2016). Learning through this system is thus effortful and controlled but evidence of learning can be found relatively fast (e.g., Carey and Bartlett, 1978). On the other hand, the procedural/implicit memory system, which is located in the basal ganglia, is assumed to underlie the implicit (non-conscious) learning and automatization of perceptual-motor as well as cognitive skills (Ullman, 2004; Ashby et al., 2010). Learning using the procedural/implicit memory system is usually unconscious as well as effortless and mostly automatic, but time and extensive repetition are required for learning to take place (e.g., Nicolson et al., 2010). In the present study, we assess the relationship between performance in tasks tapping into these two memory systems and ultimate attainment in native speakers’ linguistic knowledge in three different areas of language: grammar, vocabulary and collocations. Additionally, following from previous studies examining individual differences (e.g., Dąbrowska, 2018, 2019; Dąbrowska et al., under review), a second research question that we investigate is whether the strong relationship between lexical and grammatical knowledge which has been observed in numerous language acquisition studies survives into adulthood.

### Language and Memory in First Language Acquisition

The link between language and memory has been a recurrent research topic in recent years (Kidd and Kirjavainen, 2011; Kidd, 2012; Lum and Kidd, 2012; Lum et al., 2013; Morgan-Short et al., 2014; Hamrick, 2015; Hamrick et al., 2018). With regard to grammar, it has been almost universally assumed that first language (L1) grammatical development depends on implicit memory, and that the resulting knowledge is also almost entirely implicit (e.g., Ellis, 1996; Ullman, 2001; DeKeyser et al., 2010), in the sense that native speakers are rarely able to verbalize the grammatical rules of their language. Work on L1 acquisition has provided experimental evidence of a relationship between implicit memory and various aspects of grammatical knowledge; however, this relationship has proved to be somewhat elusive. Thus, Conti-Ramsden et al. (2015) reported a significant correlation between children’s scores in a syntactic comprehension test and their performance on a Serial Reaction Time task (SRT), which is the most commonly used measure of implicit memory for sequences (e.g., Nissen and Bullemer, 1987; Cleeremans and McClelland, 1991; Jiménez et al., 1996; Kaufman et al., 2010). Likewise, Kidd (2012) found a significant correlation between SRT and syntactic priming in a picture description task (see also Kidd and Arciuli, 2016 and Lum et al., 2012). However, several other studies have failed to find evidence of such a relationship (Gabriel et al., 2011, 2012, 2013, 2015; Lum and Kidd, 2012). A recent meta-analysis by Hamrick et al. (2018), which pooled findings from 56 correlations and 665 participants, does report a significant relationship; however, the average effect size is very small (*r* = *0.27*).

Because of the aforementioned assumption that grammar learning is mostly implicit, the potential relationship between grammar and explicit memory remains less well understood. For example, Hamrick et al. (2018) list only 3 child language studies examining this relationship, all of which show weak and non-significant correlations between grammar measures and explicit associative memory tested through verbal paired-associates recall (Kidd, 2012; Lum and Kidd, 2012; Conti-Ramsden et al., 2015; but see West et al., 2018). In spite of this, in principle, there are theoretically and empirically motivated reasons to believe that explicit memory may also play a role in L1 grammatical development. In the first place, usage-based models of language acquisition (Tomasello, 2000; Goldberg, 2006; Behrens, 2009; Bybee, 2010) assume that all linguistic knowledge is represented in the same form. As a strong relationship between explicit memory and L1 lexical knowledge has been repeatedly found (Kidd and Kirjavainen, 2011; Kidd, 2012; Lum and Kidd, 2012), if lexical and grammatical knowledge were represented in the same format, it would then follow that the explicit memory system could also contribute to the acquisition of grammatical knowledge. Secondly, this idea is partially supported by a series of studies showing significant correlations between intelligence, executive function and language aptitude, predictors mostly related to explicit learning, on the one hand, and L1 grammatical abilities, on the other (Skehan, 1989; Brooks and Kempe, 2018; West et al., 2018; Dąbrowska, 2019).

With regard to vocabulary, the common assumption is that lexical learning is supported by the explicit memory system and that vocabulary knowledge is mostly explicit (Lum et al., 2013; Hamrick et al., 2018). A very obvious argument for this is that, for example, upon hearing the word *cat*, most people will think of a miaoing quadruped and will be able to verbalize that knowledge. Older children and adults often learn the meanings of new words from their definitions, a scenario in which there is no doubt that learning and recollection are explicit. Explicit memory is also likely to be at the core of word learning through fast mapping since, in such a setting, learning of form-meaning mappings can be observed after as little as one single exposure to a given word (Carey and Bartlett, 1978; Golinkoff et al., 1992; Wilkinson and Mazzitelli, 2003). In fact, there is ample evidence that lexical knowledge relates to explicit memory, as moderately strong correlations have been reported between receptive vocabulary and verbal paired-associates recall ability (Kidd and Kirjavainen, 2011; Kidd, 2012; Lum and Kidd, 2012; Lum et al., 2012).

Nevertheless, implicit memory may also be relevant for vocabulary learning. Studies on cross-situational learning have repeatedly shown that form-meaning mappings can be learned by unconsciously tracking co-occurrence patterns for objects and their labels (Smith and Yu, 2008; Smith et al., 2011; Yurovsky and Frank, 2015). As a matter of fact, cross-situational learning has been suggested by some as the mechanism through which children acquire their L1 vocabulary (Pereira et al., 2013; Vlach and Johnson, 2013). Furthermore, learning new words may also involve the implicit tallying of collocational patterns for specific words (Dąbrowska, 2009). In spite of this, although several studies have probed the link between lexical knowledge and implicit memory, none has found a significant relationship between the two (Gabriel et al., 2011, 2012, 2013, 2015; Kidd and Kirjavainen, 2011; Kidd, 2012; Lum et al., 2012).

Finally, little is known about the mechanisms underlying the learning and storage of collocations. By collocations, we mean combinations of words that conform to the rules of grammar and have compositional meanings, but occur together more frequently than it would be expected from the frequencies of the words themselves (e.g., *boost production* or *heavy traffic;* Dąbrowska, 2014). A reasonable assumption put forward by some L2 researchers (e.g., Forsberg Lundell and Sandgren, 2013), is that the learning of collocations relies on the implicit memory system because it results from the implicit tallying of co-occurrences of words in speech and texts. In line with this idea is the fact that native speakers are generally not aware of collocational restrictions unless these are violated. Partial evidence in support for this hypothesis for L1 learners has been provided by Yi (2018), who found that processing speed for multi-word sequences in the L1 is predicted by one’s implicit memory as measured by an SRT task.

Another memory system which is relevant to language learning is phonological short-term memory (PSTM), or the phonological loop. Speech is fleeting; and the phonological loop is a buffer in which incoming information is maintained before it can be permanently encoded in long-term memory (Baddeley et al., 1998). Phonological short-term memory is an explicit system in the sense that the learner is aware of its content and often intentionally attempts to maintain it in memory (e.g., by subvocal rehearsal); and PSTM measures are known to correlate with IQ.

It has been proposed that the phonological loop is basically a language learning device (cf. Baddeley et al., 1998). This hypothesis is consistent with the fact that impairments of phonological memory in children are associated with language delay (Gathercole and Baddeley, 1990b; Gray, 2003; Gathercole, 2006; Estes et al., 2007) and that adults with acquired phonological memory deficits are unable to learn the phonological forms of new words (Baddeley et al., 1988; Trojano and Grossi, 1995). Furthermore, a number of studies have demonstrated the existence of robust correlations between phonological memory measures such as non-word repetition and vocabulary size and/or speed of vocabulary learning in young children (Gathercole and Baddeley, 1989, 1990a; Adams and Gathercole, 1996, 2000; Gathercole et al., 1997) and L2 learners at lower levels of proficiency (Cheung, 1996; Atkins and Baddeley, 1998). There is some evidence that such a relationship may also be present in older children and adolescents and advanced L2 learners; however, the evidence for this is less consistent, with many studies failing to find such a relationship (see Hummel, 2009 and Cheetham, 2014 for reviews). This is most likely due to the fact that speakers with larger vocabularies can use long-term memory representations to support phonological encoding while processing novel words. Some studies have also found that performance on phonological memory tasks correlates with learner’s knowledge of syntax; however, the evidence for this relationship is less consistent than the evidence linking PSTM to vocabulary learning (cf. Hummel, 2009), and the relationship may be mediated by vocabulary (Service and Kohonen, 1995).

Phonological short-term memory can be measured using digit, letter or word span tasks, which involve participants repeating series of digits, letters or words of increasing length. An alternative method involves using non-word repetition or recognition: participants are asked to either repeat sequences of nonsense syllables or to decide whether two sequences of nonsense syllables are the same or different (Gathercole et al., 1999). Gathercole et al. (1997) argue that non-word repetition is a purer measure of PSTM than digit span because it requires the participant to attend to subtle phonological distinctions and develop finer-grained representations of the stimuli, while performance on digit span is more affected by prior linguistic knowledge. In their data, non-word repetition was more strongly associated with vocabulary size than digit span, and the relationship was more specific. Non-word repetition tasks also discriminate better between typically developing children and children with language impairment (Gray, 2003; Gathercole, 2006). On the other hand, digit span tasks require the participant to encode longer stretches of text, and this characteristic may make it a better predictor of the ability to learn multi-word units such as collocations and grammatical patterns. Furthermore, to the extent that later vocabulary learning relies more on extracting information from the linguistic as opposed to the extra-linguistic context, the digit span may be a more relevant predictor of later vocabulary learning. For this reason, in this study we use a digit span task as a measure of phonological memory.

### The Link Between Grammar, Vocabulary and Collocations

The second goal of the present paper is to provide a clear characterization of the relationship between the ultimate attainment of grammar, vocabulary and collocational knowledge. This is a theoretically important issue because modular and usage-based models of language acquisition make diverging predictions about the relationship between grammar and vocabulary. Modular models (Chomsky, 1965; Pinker, 1994, 1999; Ullman et al., 1997; DeKeyser et al., 2010) assume that grammar and vocabulary are learned through fundamentally different mechanisms and rely on the two main memory systems differently. Thus, according to these models, there is no *a priori* reason to predict a strong relationship between grammatical and lexical knowledge except perhaps in early stages of acquisition, where a critical mass of lexical items may be required to trigger grammatical learning and, conversely, knowledge about the syntactic contexts in which words occur may bootstrap lexical acquisition. By contrast, usage-based models (Tomasello, 2000; Langacker, 2009; Bybee, 2010) and related frameworks such as emergentism (e.g., MacWhinney, 2015) and connectionism (e.g., Rumelhart and McClelland, 1986; Elman et al., 1996), assume that all linguistic knowledge is acquired using the same mental mechanisms and represented in the same format. Therefore, all of these approaches predict a relatively strong relationship between grammatical and lexical knowledge.

There is a large amount of evidence supporting the existence of a robust relationship between grammatical and lexical development in first language acquisition (Bates et al., 1988; Caselli et al., 1999; Marchman et al., 2004). For example, Bates et al. (1988) found that children’s use of grammatical morphemes at 20 months of age was strongly correlated with their total vocabulary (*r* = 0.87). In addition, these authors found that vocabulary size at 1;8 years of age predicted mean length of utterance (MLU) at 2;4 years even better than MLU at 1;8 years. Nonetheless, these results in early L1 acquisition have more than one possible explanation. While it can indeed be the case that vocabulary and grammar development are strongly related across the board, an alternative explanation is that the relationship is especially strong in early L1 acquisition because vocabulary paces grammatical development: that is, grammatical development is constrained by vocabulary learning because grammatical patterns can only be extracted from the input if the words are known in the first place (Marchman and Bates, 1994; Hoff et al., 2018). Note, however, that recent evidence suggests that the relationship between grammatical and lexical knowledge also holds for adult speakers (Dąbrowska, 2018; Dąbrowska et al., under review), although it may be weaker than for children (*r* = 0.40 in Dąbrowska, 2018).

Finally, there is little research examining whether (and if so, how) knowledge of collocations relates to other aspects of language. There is some evidence, however, that collocational knowledge is indeed related to both grammatical knowledge and vocabulary. Riches et al. (under review) report significant correlations between tasks probing knowledge of collocations and grammar (*r* = 0.51) and collocations and vocabulary (*r* = *0.50*) in children learning their native language. Similarly, Dąbrowska (2018) observed a significant correlation between collocations and grammar (*r* = *0.36*) and an even stronger one between collocations and vocabulary (*r* = 0.57) for adult speakers. A close relationship between collocations and grammar fits well with the idea put forward by Forsberg Lundell and Sandgren (2013) that collocational learning relies on the implicit tallying of co-occurrence statistics, which is also the mechanism said to underlie native grammar acquisition (e.g., Ellis, 1996; Ullman et al., 1997). The strong relationship between collocations and vocabulary outlined above, on the other hand, goes hand in hand with the proposal that vocabulary learning from mid-childhood onward depends on tracking co-occurrence patterns between specific words and their collocates (e.g., Dąbrowska, 2009).

### The Present Study

In the present study, we investigate the relationship between ultimate attainment in grammar, vocabulary and collocations on the one hand, and implicit and explicit memory on the other. Native speakers of English were tested in three language tasks, each concerned with one specific language area, and three memory tasks assessing implicit memory for sequences, explicit memory for cross-modal associations, and phonological short-term memory span. Measures obtained from the memory tasks were subsequently used as predictors of accuracy in the language tasks.

The vast majority of psychological and psycholinguistic research is conducted with university students and graduates, which raises the concern that the results may not be generalizable to other populations (Henrich et al., 2010; Jones, 2010; Rad et al., 2018). To avoid this possible limitation, we recruited participants from two different educational backgrounds and explicitly compared their performance. This is especially relevant for the questions examined in the present study because academic attainment has been shown to modulate performance on a variety of linguistic tasks (Street and Dąbrowska, 2010; Street, 2017; Dąbrowska, 2018). In spite of this, it still remains unclear whether the effects of academic attainment on linguistic knowledge are attributable to differences in education itself or other factors that correlate with education, such as print exposure. In order to shed some light on this issue, we also collected information about participants’ reading habits.

Based on previous results, and in agreement with usage-based models of language acquisition (Tomasello, 2000; Langacker, 2009; Bybee, 2010), we predict that all three memory measures will be associated with knowledge of grammar, vocabulary and collocations, although not necessarily to the same degree. This is because we assume that all linguistic knowledge is represented in the same format (i.e., form-meaning pairings, or constructions) and acquired by means of the same cognitive mechanisms. For the same reason, we additionally expect performances on the three language tasks to be strongly correlated.

## Materials and Methods

### Participants

Sixty native speakers of English took part in this study in exchange for a small payment. All participants gave their informed consent to participate and the study was conducted in accordance with the Declaration of Helsinki. Forty participants (29 females; mean age = 21.63, *SD* = 0.81) were university students recruited at several campuses around the United Kingdom. This group will be henceforth referred to as High Academic Attainment (HAA). The remaining 20 participants (2 females; mean age = 19.65, *SD* = 2.39) were vocational students recruited at United Kingdom colleges offering vocational training. This group will be referred to as Low Academic Attainment (LAA). All participants filled in a background questionnaire in which they provided basic demographic information as well as information about their reading habits.

### Materials

This study consisted of three cognitive tasks and three language tasks. Tasks are described one by one below in the order in which they were presented to participants.

#### Forward Digit Span Task

Digit span is the standard test of phonological short-term memory used in psychological studies and assessment batteries (e.g., Richardson, 2007; Wechsler, 2008). In our version of the task, sequences of numbers were presented on the screen, one number at a time, and participants were subsequently asked to recall the numbers in their order of appearance. Each number stayed on the screen for 1000 ms and the interstimulus interval was 300 ms. The initial sequences were three digits long (e.g., 397) and length increased gradually until reaching nine digits (e.g., 214569821). All numbers from 0 to 9 were possible and so were repetitions of the same numbers within each sequence. There were three sequences of each length (i.e., from three to nine) for a total of 21 trials. Participants responded using the numeric keys on the computer keyboard.

### Serial Reaction Time Task

The serial reaction time (SRT) task is commonly used to examine implicit memory for sequences (e.g., Nissen and Bullemer, 1987; Cleeremans and McClelland, 1991; Jiménez et al., 1996; Kaufman et al., 2010). Our task was adapted from the High-Level Language Aptitude Battery (Hi-LAB; Linck et al., 2013). Participants were seated in front of a computer screen which showed four underscores (_). On each trial, an asterisk (^∗^) appeared right above one of the underscores, and the participants’ task was to press the corresponding button of the response box (labeled from 1 to 4) as quickly and accurately as possible. 500 ms after the button press, the asterisk appeared in a different position. The task was divided into 6 blocks of 96 trials each. In the first and last blocks, the positions in which the asterisk appeared were determined in a pseudorandom order, while in blocks 2, 3, 4, and 5, the asterisk appeared in a repeating sequence of length 12 (i.e., 1–2–1–4–3–2–4–1–3–4–2–3).

Some researchers (e.g., Kaufman et al., 2010; Granena, 2013; Suzuki and DeKeyser, 2015) have argued that probabilistic SRT tasks, in which the sequence of stimuli is generated by a probabilistic rule, provide a better measure of implicit memory than the traditional deterministic task used here because the deterministic variant may be influenced by explicit learning during the task. However, we decided to use the traditional version of the task because it has been found to be more reliable than the probabilistic version and reliability was an important concern due to our individual differences approach. For example, Granena (2018) and Granena and Yilmaz (2018) both report a split-halves reliability of 0.79 for deterministic SRT tasks, while split-halves reliabilities for the probabilistic SRT tasks in Kaufman et al. (2010); Granena (2013) and Suzuki and DeKeyser (2015) are 0.33, 0.44 and 0.42, respectively. The potential influence of differences between SRT variants on the results reported here will be further taken up in the discussion.

#### Paired-Associates Task

As a measure of explicit memory for cross-modal associations, we used LLAMA-B, a computerized paired-associates test from the LLAMA Language Aptitude Tests (LLAMA-B; Meara, 2005). LLAMA-B is a vocabulary learning task that is loosely based on the paired-associates test in the Modern Language Aptitude Test (Carroll and Sapon, 1959). Participants were presented with 20 target images, each of which corresponded to a word from a Central American language, and were given two minutes to study the word-picture associations. In the subsequent test phase, the twenty words were displayed one by one and, for each of them, participants had to identify the correct picture on the screen.

#### Grammar

Grammatical proficiency was examined by means of a computerized, written grammaticality judgment task (GJT). The items used in this task were a subset of those used in DeKeyser (2000) and Johnson and Newport (1989). They were 60 English sentences, of which 30 were grammatical and the other 30 contained a grammatical error. DeKeyser examined the performance of L2 learners on English sentences targeting 10 different aspects of grammar that are often problematic for non-native speakers (e.g., past tense, plural marking, third-person singular, etc.). When selecting the stimuli, we made sure that our task included a balanced number of grammatical and ungrammatical items for each of the different features. A list of the items used in our study is provided in the Supplementary Materials.

Participants were told that they would be visually presented with English sentences and that their task would be to assess whether the sentences were possible/accurate/proper English sentences or not. In each trial, a sentence was presented in the middle of the screen for 5000 ms and then disappeared. This was done in order to prompt relatively fast responses from participants^2^. Together with the sentence, two small boxes appeared at either side of the screen: a green one with “correct” written on it on the left and a red one with “incorrect” on the right. Participants were instructed to press the leftmost key of the response box (with a green key top) if they considered the sentence to be correct, and the rightmost key (with a red key top) if they considered it not to be correct. Note that the keys to be pressed matched the boxes visually presented in color and spatial location.

#### Collocations

Knowledge of English collocations was measured using a computerized version of the Words that Go Together Test (Dąbrowska, 2014). This test contains 40 multiple-choice items, each consisting of five short phrases. The participants’ task was to choose, in each case, the phrase that they considered to look “the most natural or familiar.” Target phrases were adjective-noun and verb-noun collocations (e.g., *blatant lie*, *raise prices*; see Dąbrowska, 2014 for further details), while the four distractor phrases for each trial were semantically similar adjective-noun or verb-noun combinations with lower mutual information scores (e.g., *clear lie*, *grow prices*). Participants were presented with the five phrases preceded by the numbers 1 to 5 on the computer screen and were asked to press the button on the response box corresponding to their response.

#### Vocabulary

A computerized implementation of the revised and re-standardized vocabulary component of the Shipley Institute of Living Scale (Shipley-2; Shipley et al., 2009) was used as English vocabulary test. This is a multiple-choice receptive-vocabulary test consisting of 40 English words of differing lexical frequencies. In each trial, one English word was presented on the screen in uppercase letters (e.g., MASSIVE) and four possible responses were provided in lowercase preceded by the numbers 1 to 4 (e.g., 1. bright, 2. large, 3. speedy, 4. low, “large” being the correct answer). Participants had to press the button of the response box (labeled from 1 to 4) corresponding to the word whose meaning was most similar to that of the uppercase word.

### Procedure

Participants were tested individually or in groups of two in a quiet room at the university or college where recruitment took place. The experimental session took approximately 45 min. The testing station consisted of a Lenovo Thinkpad L580 laptop with a 15.6-inch screen, a seven-button Cedrus RB-740 response box and a set of noise-reducing headphones. The paired-associates task was administered using the software that comes with the LLAMA Test. All remaining tasks were created and run using Psychopy3 (v. 3.0.2; Peirce et al., 2019).

### Data Analysis

Individual values for each task were extracted before conducting any statistical analyses. For the digit span task (henceforth Digit Span), paired-associates task (henceforth LLAMA-B) and the three language tasks, individual scores were calculated as the proportion of correct responses provided by each participant. For the serial reaction time task (henceforth SRT), two scores were calculated. The first was computed using the traditional method, i.e., by subtracting the mean RT for correct trials in the final sequential block (5) from the final random block (6). Therefore, higher scores were indicative of stronger effects of implicit sequence recollection. This measure will be henceforth referred to as SRT (traditional). The second score was based on the slope of the log-transformed RT function over the 4 sequential blocks (i.e., blocks 2, 3, 4, and 5) for each participant. This measures how much RT decreased as a function of exposure to the repeating sequence. SRT slopes were calculated following the procedure outlined in Divjak and Milin (2020). On this measure, lower (i.e., more negative) scores correspond to a steeper decrease in RTs and hence evidence a stronger impact of exposure to the sequence. Finally, we calculated a measure of print exposure, the Reading Index, on the basis of participants’ responses to two questions in the background questionnaire. The Reading Index was the mean of responses to the questions “How much do you read in a typical week?” and “How much did you read last week?”. Responses were given on a scale going from 1 to 6, where 1 corresponded to “less than one hour,” 2 to “1–5 h,” 3 to “6–10 h,” 4 to “11–15 h” and 6 to “more than 20 h”.

## Results

Data from two participants, one from each group, for the Digit Span task were missing due to computer malfunction, as well as data from one LAA participant for the Vocabulary task and one for SRT. In addition, results for one LAA participant in the Grammar task and another LAA participant in the Collocations task were excluded because their responses were abnormally quick. They showed more than 5 consecutive trials with RTs shorter than 1000 ms and average RTs over the whole task shorter than 1500 ms and therefore much shorter than the mean RT for these tasks (mean RT for Grammar = 2717 ms; Collocations = 5314 ms). This strongly suggested that they had not fully engaged with the task. Table 1 shows the means and standard deviations for all tasks for the two groups as well as for all participants pooled together. Comparisons via Welch two sample *t*-tests, in order to account for the samples’ unequal variances (Welch, 1947), indicated that participants in the HAA group outperformed participants in the LAA group in all experimental tasks except for the SRT task, where there is only a marginally significant difference for slopes and a non-significant difference for the traditional SRT measure. In addition, Reading Indices were also higher for the HAA than the LAA group.

**TABLE 1:** Means and standard deviations (in parentheses) for all measures by group and with the two groups pooled together.

Group	HAA	LAA	All	Comparison HAA vs. LAA
Grammar (%)	91.5 (4.5)	72.1 (11.5)	85.3 (11.7)	*t*(20.66) = 7.13, *p* < 0.001
Vocabulary (%)	78.6 (8.5)	54.3 (11.6)	70.8 (14.9)	*t*(27.68) = 8.15, *p* < 0.001
Collocations (%)	71.8 (11.1)	42 (13.5)	62.2 (18.3)	*t*(30.01) = 8.39, *p* < 0.001
Digit Span (%)	59 (14.7)	42.4 (15.9)	53.5 (16.9)	*t*(33.48) = 3.83, *p* < 0.001
LLAMA-B (%)	60.9 (20.2)	27.5 (19.2)	49.8 (25.3)	*t*(39.88) = 6.23, *p* < 0.001
SRT (traditional; ms)	22.4 (40.6)	3 (85.9)	16.2 (59)	*t*(21.9) = 0.94, *p* = 0.36
SRT (slope)	−0.00057 (0.0010)	0,000021 (0.0012)	−0.00037 (0.0011)	*t*(33.34) = −1.92, *p* = 0.06
Reading Index (1-6)	3.7 (1.3)	1.9 (0.9)	3.1 (1.5)	*t*(52.14) = 6.33, *p* < 0.001

*Individual scores for all tasks except SRT are presented as percentages correct for ease of interpretation. Statistical differences were calculated by means of Welch two sample *t*-tests.*

So as to get a first impression of the relationships between language and memory tasks, as well as relationships between tasks within each set, we computed simple correlations between all the measures. These are presented in Table 2. Three facts are immediately observable on inspecting the table. First of all, there are very strong relationships (*rs* ranging from 0.70 to 0.82) between the three language tasks. Secondly, performance on all three language tasks is robustly correlated with phonological short-term memory as measured by the Digit Span task (*r*s ranging from 0.56 to 0.64) and associative memory as measured by LLAMA-B (*r*s from 0.61 to 0.65). Weaker but nevertheless significant correlations are also observed between one of the measures of implicit memory, namely the slope of the decrease in RTs, and accuracy in the vocabulary and collocations tasks (*r*s of −0.34 and −0.28, respectively). Finally, all three language tasks also show small- to medium-sized correlations with the Reading Index (*rs* from 0.32 to 0.48).

**TABLE 2:** Correlation matrix for the three language tasks (Grammar, Vocabulary, and Collocations), the three memory tasks [Digit Span, LLAMA-B, SRT (traditional + slope)] and Reading Index for all participants together.

	Gr	Voc	Coll	DS	LL-B	SRT (t)	SRT (s)	Read
Gr	1.00							
Voc	0.70***	1.00						
Coll	0.80***	0.82***	1.00					
DS	0.64***	0.56***	0.60***	1.00				
LL-B	0.65***	0.63***	0.61***	0.44***	1.00			
SRT (t)	0.12	0.18	0.20	0.23	0.11	1.00		
SRT (s)	–0.20	−0.34**	−0.28*	−0.31*	–0.13	−0.56***	1.00	
Read	0.32*	0.48***	0.35**	0.17	0.29*	0.20	−0.35**	1.00

*significant at *p* < 0.05, **significant at *p* < 0.01, ***significant at *p* < 0.001.

After this first correlational analysis, data were submitted to further analyses in order to gain more detailed insights on the aforementioned relationships. Missing values in the dataset consisting of individual scores per measure were imputed by means of productive mean matching using the *mice* package (van Buuren and Groothuis-Oudshoorn, 2011) in R (version 3. 5. 2; R Core Team, 2017). Subsequently, a linear mixed-effects regression model was fit (*lme4* package 1.1-20; Bates et al., 2015) with Accuracy (i.e., proportion correct responses for the three language tasks^3^) as dependent variable. Categorical independent variables included Language Task (Grammar/Vocabulary/Collocations) and Group (HAA/LAA).

Language Task was factor coded with Grammar as the reference level. Group was contrast coded such that LAA was −0.5 and HAA corresponded to 0.5 and was therefore treated as numeric in the analysis. Other numeric independent variables included Digit Span (phonological short-term memory), LLAMA-B (explicit associative memory), SRT Slope (implicit memory)^4^ and Reading Index. These four predictors were centered and scaled. Two-way interactions between Group and Task, as well as between Task and each of the four numeric independent variables, and Group and each of the four numeric predictors were also included. These were necessary in order to assess whether the effects of Digit Span, LLAMA-B, SRT slope and Reading Index differed as a function of the language task on the one hand and as a function of the academic attainment of participants on the other. Finally, we also included three-way interactions between Task, Group and each of the four numeric independent variables because these allowed us to elucidate more complex relationships between predictors (e.g., whether differences in the influence of LLAMA-B between the three language tasks were additionally modulated by group). Random intercepts by participant were included to account for the fact that there were 3 values per participant in the dataset (i.e., Grammar, Vocabulary, and Collocations accuracies). The significance of main effects and interactions was assessed using hierarchical partitioning of the variance via nested model comparisons. This means that the full model was fitted first, and then predictors were removed one by one, beginning with the highest-level interaction terms. If the absence of a particular predictor resulted in the model exhibiting a worse fit to the data, as determined through log-likelihood ratio tests, the predictor was considered to have a significant effect and was kept in the model. If it did not, the predictor was removed and the procedure started again for the next predictor.

**FIGURE 1:**
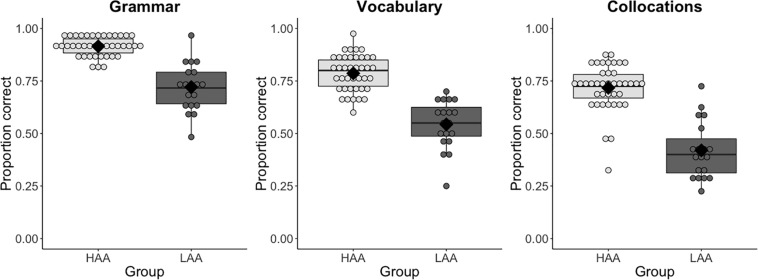
Proportion of correct responses in the Grammar (left), Vocabulary (center), and Collocations (right) tasks by Group (LAA – in dark gray; HAA – in light gray). Dots showcase the distributions of individual values within each group and the black diamonds signal group means.

**FIGURE 2:**
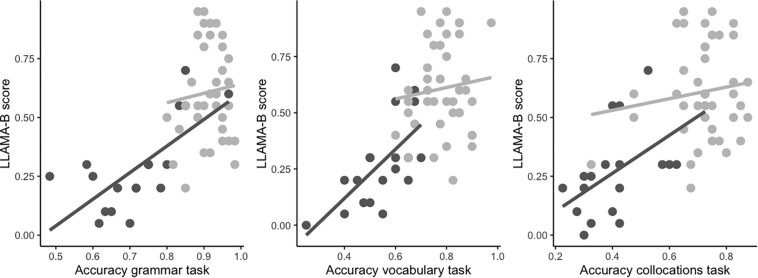
Scatterplots of individual values for LLAMA-B scores and individual scores for Grammar (left), Vocabulary (center), and Collocations (right) by Group (LAA – in dark gray; HAA – in light gray). Regression lines for each group are provided for illustration purposes.

Model comparisons revealed significant main effects of Language Task [χ*^2^* (2) = 148.01, *p* < 0.001], Group [χ*^2^* (1) = 25.06, *p* < 0.001], Digit Span [χ*^2^* (1) = 12.43, *p* < 0.001] and LLAMA-B [χ*^2^* (1) = 11.98, *p* < 0.001]. The coefficients and significance levels of all predictors in the best-fitting model are reported in Table 3. These results indicate, in the first place, that participants’ accuracy differed between the three language tasks and that participants in the LAA group were overall less accurate than participants in the HAA group. This is observable in Figure 1, which shows both group and individual accuracy values per task per group. Secondly, our results show that accuracy in the language tasks was modulated by scores in the Digit Span and LLAMA-B tasks: the higher the Digit Span and LLAMA-B scores, the higher the accuracy in the language tasks. In addition to this, the model also revealed significant two-way interactions between Language Task and Group [χ*^2^* (2) = 9.64, *p* < 0.01] and Group and LLAMA-B [χ*^2^* (1) = 6.99, *p* < 0.01]. The interaction between Language Task and Group indicates that differences in accuracy between the groups varied depending on the task examined, with the collocations task showing the largest group differences (see Figure 1). The interaction between Group and LLAMA-B further shows that the influence of LLAMA-B on linguistic proficiency differed for the two groups, being stronger for the LAA group than for the HAA group. This difference is apparent in Figure 2, which showcases the relationship between individual LLAMA-B scores and accuracy for each of the three language tasks for the two groups of participants. Crucially, the fact that there were no interactions between Language Task and Digit Span [χ*^2^* (2) = 2.15, *p* = 0.34], Language Task and LLAMA-B [χ*^2^* (2) = 0.37, *p* = 0.83] or three-way interactions involving Language Task, Group and one of the two explicit memory predictors (both *p* > 0.5) indicates that the effects of Digit Span and LLAMA-B do not differ significantly across linguistic domains. Additional least-squares regression analyses with data split by task and all significant variables in the main analysis as predictors are provided in the Supplementary Materials to further illustrate the consistency of results across tasks.

**TABLE 3:** Coefficients and significance values for the final, best fitting linear mixed-effects regression model assessing the effects of Task (Grammar, Vocabulary, and Collocations), Group (HAA and LAA), Digit Span, LLAMA-B, SRT (slope), and Reading Index on accuracy in the language tasks.

Predictor	*b*	*t*	*p*
Intercept	0.85	58.06	<0.001
Task (vocabulary)	–0.15	–9.59	<0.001
Task (collocations)	–0.25	–15.74	<0.001
Digit Span	0.03	3.71	<0.001
LLAMA-B	0.04	3.47	<0.01
Group	0.92	3.10	<0.01
Task (vocabulary) x Group	0.04	1.35	0.18
Task (collocations) x Group	0.10	3.11	<0.01
LLAMA-B x Group	–0.06	–2.78	<0.01

*Significance of variables was assessed by means of Satterthwaite’s approximation for degrees of freedom using the lmerTest package (Kuznetsova et al., 2017).*

## Discussion

The present study investigated the relationship between explicit and implicit memory on the one hand and ultimate L1 language attainment in the areas of grammar, vocabulary and collocations on the other. We also examined the potential modulating role of academic attainment and print exposure in these relationships and the relationships between grammar, vocabulary and collocations scores. The following discussion is divided into sections addressing (i) the effects of academic attainment and reading, (ii) the effects of explicit memory, (iii) the lack of robust effects of implicit memory, and (iv) the relationship between the three language tasks.

### Academic Attainment and Reading

Our results showed a strong effect of academic attainment on participants’ performance on both language and cognitive tasks. Participants in the HAA group outperformed those in the LAA group in all three language tasks. These group differences replicate the findings of several earlier studies which demonstrated a strong link between language and academic attainment (Dąbrowska, 1997, 2018; Chipere, 2003; Street and Dąbrowska, 2010; Street, 2017). Furthermore, effects of academic attainment were also observed for the digit span task assessing explicit, phonological short-term memory and LLAMA-B, which examined participants’ ability to explicitly establish cross-modal associations^5^. Differences between groups for the digit span task are not surprising, as this measure is often included in IQ test batteries, and academic achievement is known to correlate strongly with IQ (e.g., Wechsler, 1958; Kaufman and Lichtenberger, 2006; Deary et al., 2007; Hogan et al., 2010). Likewise, the differences observed for LLAMA-B fit well with previous research showing that explicit memory for cross-modal associations correlates with reading achievement^6^ and other factors related to educational success (Vellutino et al., 1975; Poulsen and Elbro, 2018; Ehm et al., 2019). Thus, the differences between high and low academic attainment participants are not necessarily attributable to education as such, but could be due to other factors that are known to correlate with academic attainment^7^.

The results for the SRT task are less conclusive. When implicit memory was assessed using the traditional measure (i.e., by subtracting the RTs for the last sequential block from those for the final random block), the HAA group showed a modest benefit of exposure to the sequence (22.4 ms) that was similar to those observed in some previous studies (20–40 ms; Granena, 2018; Granena and Yilmaz, 2018; Yilmaz and Granena, 2019). The LAA participants, however, showed virtually no changes when their performance was assessed as a group (3 ms). However, as discussed earlier, the group difference is not statistically significant. A similar pattern can be seen when performance is assessed using the slope measure, which, according to Divjak and Milin (2020), is more sensitive: participants in the HAA groups showed, as expected, negative slopes (i.e., their RTs got shorter as the task progressed), whereas the LAA group had an average slope that was very close to zero (and in fact positive). In this case, the between-group comparison was marginally significant.

Clearly, further research is necessary to determine whether this apparent group difference is real. The finding is surprising, since implicit memory for sequences has repeatedly been shown not to relate to factors which are known to be strongly tied to academic attainment, such as explicit learning abilities and intelligence (Reber et al., 1991; Maybery et al., 1995, Gebauer and Mackintosh, 2007). However, most studies which used SRT to measure implicit memory for sequences in adults tested HAA participants (e.g., Stefaniak et al., 2008; Romano et al., 2010; Hamrick, 2015; Faretta-Stutenberg and Morgan-Short, 2018). If individual differences in implicit memory are indeed related to academic attainment, these findings may then not generalize to other populations.

Regarding the potential influence of print exposure and reading practices on L1 ultimate attainment, the individual self-reported reading measure used in the present study showed significant correlations with all three language tasks but its effect failed to reach significance in the subsequent regression analyses. The lack of an effect in the latter is at odds with some earlier studies. For instance, Dąbrowska (2018) reports a small but significant contribution of print exposure to predicting scores in a task assessing grammatical knowledge, and much stronger effects for vocabulary and collocation scores. For all three tasks, the effects were observed over and above the effects of IQ and educational attainment. Likewise, Stanovich (1986); Cunningham and Stanovich (1998), and many others have found robust correlations between print exposure and vocabulary. The most likely explanation for this difference is that the self-reported measure used here is not as reliable as the objective measures used in these earlier studies, and hence more power would be required to detect the effect.

### Explicit Memory

Our results show strong and significant correlations between the two explicit memory measures, namely phonological short-term memory (measured by the forward Digit Span task) and memory for cross-modal associations (measured by LLAMA-B), and scores on all three language tasks. Subsequent mixed-effects regression analyses provided additional insights into these results by showing that performance on the Digit Span task predicted performance on the language tasks for both groups of participants, while LLAMA-B scores robustly predicted accuracy for the LAA group only.

The effect of phonological short-term memory adds to previous research emphasizing the relevance of this type of memory for vocabulary acquisition in L1-learning children (Gathercole and Baddeley, 1990a; Adams and Gathercole, 1996, 2000; Verhagen and Leseman, 2016) and L2 learners at lower levels of proficiency (Cheung, 1996; Atkins and Baddeley, 1998). Similarly, this is also in line with the findings of previous research linking PSTM to grammar acquisition in early L1 acquisition (Blake et al., 1994; Adams and Gathercole, 1995). As mentioned in the introduction, earlier research has found that effects of PSTM were much more consistent for vocabulary than for grammar, and in younger learners than in older learners. To our knowledge, our study is the first to find robust effects of PSTM in adult native speakers. It is noteworthy that we found effects of a similar magnitude for all three areas of language we studied, including collocations, which has received little attention from this perspective. This is most likely due to the fact that the digit span task requires participants to maintain longer stretches of text, and thus the phonological encoding thereof, in short-term memory and is therefore more relevant for aspects of language which involve syntagmatic relations, namely phraseology and syntax. The relationship between digit span and vocabulary knowledge may be explained by the fact that later vocabulary development depends to a large extent on learning from the linguistic contexts in (primarily written) texts (Dąbrowska, 2009).

Regarding explicit memory for cross-modal associations, the fact that performance on LLAMA-B predicted vocabulary attainment, even if only for LAA participants, fits well with studies which found a relationship between explicit associative memory and L1 lexical development (Kidd and Kirjavainen, 2011; Kidd, 2012; Lum and Kidd, 2012; Lum et al., 2012). The significant relationship between associative memory and grammar found in the LAA group, however, contrasts with the findings of several studies which failed to find a relationship between the two (Kidd, 2012; Lum and Kidd, 2012; Conti-Ramsden et al., 2015; see, however, Kidd and Kirjavainen, 2011). Importantly, as it will be further discussed below, the fact that grammar and vocabulary show similar relationships with explicit memory can be seen as support for the usage-based view that lexical and grammatical knowledge are represented in the same format (Tomasello, 2000; Goldberg, 2006; Behrens, 2009; Bybee, 2010).

As we have seen, LLAMA-B predicted performance on the language tasks for participants with lower academic attainment but not for the HAA group. It should be stressed that the lack of an effect in the HAA group cannot be attributed to ceiling effects. As shown in Figure 2, HAA participants’ scores on LLAMA-B range from 0.2 to 1 and there is a visible spread of values between the two ends. It is true that there is less variation in performance on the language tasks, particularly on grammar. However, the fact that Digit Span shows comparable effects for both groups of participants suggests that there is sufficient variation in scores in the HAA group to show an effect, if there is one. An alternative explanation for the interaction between LLAMA-B and group is that the relationship between explicit associative memory and language is not strictly linear, but instead, associative memory only affects language attainment up to a certain level. Note that scores for more than half of LAA participants were below the lowest score for HAA. Therefore, it could be the case that better memory for cross-modal associations aids language learning only in individuals who achieve relatively low scores on this memory measure.

Another finding that is worth noting is that both of our explicit memory tasks correlated more strongly with the language tasks (Digit Span: *r* from 0.56 to 0.64; LLAMA-B: *r* from 0.61 to 0.65) than with each other (*r* = 0.44). In fact, comparisons of correlations from dependent samples via Fischer’s *r*-to-*z* transformations followed by an asymptotic z-test (Steiger, 1980; Lee and Preacher, 2013) show that, apart from the correlation between Digit Span and Vocabulary (*z* = 1.26, *p* = 0.10), all other correlations between the two explicit memory measures on the one hand and the language tasks on the other are significantly higher (*z* > 1.6, *p* < *0.05*) than the correlation between Digit Span and LLAMA-B. This somewhat puzzling pattern of correlations may be due to the fact that phonological short-term memory and longer-term memory for cross-modal associations support language learning in rather different ways. Explicit memory for phonological sequences makes it possible for the learner to acquire syntagmatic associations. These are obviously relevant for grammar and collocations, and, to the extent that learning word meanings depends on tracking co-occurrence patterns in texts (cf. Dąbrowska, 2009), they could also support vocabulary learning. The ability to form cross-modal associations, on the other hand, helps with learning sound-meaning correspondences. These are obviously relevant for vocabulary learning, but, in a usage-based model, also for grammar and collocations, which according to this model are also represented as form-meaning pairings.

### Implicit Memory

Our results did not show a robust relationship between implicit memory (assessed using an SRT task) and ultimate attainment in the native language. While weak but significant correlations between SRT slopes and vocabulary and collocations (but not grammar) were observed, the effect of this predictor failed to reach significance in a linear mixed-effects regression analysis. Our failure to find a significant relationship does not, of course, mean that implicit memory is not relevant to language acquisition. As pointed out in the introduction, the results of previous research are mixed: while a number of studies have found such a relationship, there are also many that did not. As briefly discussed in West et al. (2018), it is possible that this is due to the fact that SRT tasks are simply not very reliable.

However, there are also three additional possible explanations for the lack on an effect in the present study that do not necessarily stem from task unreliability. A first possibility is that our sequential or deterministic SRT task could in principle have been influenced by explicit memory to a certain extent, which could have distorted the results. However, we consider this rather unlikely in the light of the findings that (i) participants in the LAA group show very little learning when examined as a group, and (ii) explicit memory was found to significantly predict performance in the language tasks. Particularly, point (ii) suggests that, if explicit memory had indeed played a role in the SRT task, then it may have boosted the predictive power of SRT rather than reduced it, and yet SRT was not found to relate to performance in the language tasks. The second possibility is that the role of implicit memory changes over the course of development, being more relevant in the earlier developmental stages than in adulthood. Critically, this hypothesis is in line with the fact that most research addressing the link between implicit memory for sequences and language and showcasing a relationship between the two were run on children (Kidd, 2012; Lum et al., 2012; Conti-Ramsden et al., 2015; Kidd and Arciuli, 2016), while the existence of such a relationship for adult L1 speakers is less clear (but see Misyak et al., 2010). Finally, a third non-exclusive alternative is that the effect is simply hard to find because true individual differences in implicit memory are small, and hence would require more statistical power to be detected.

### The Relationship Between Grammar, Vocabulary, and Collocations

Lastly, we found very strong correlations between participants’ scores in the grammar, vocabulary and collocations tasks. The tight connection between grammar and vocabulary in adult L1 speakers reported here goes hand in hand with the results of recent studies (Dąbrowska, 2018; Dąbrowska et al., under review) indicating that this relationship, well documented for L1-learning children (Bates et al.,1988; Caselli et al., 1999; Marchman et al., 2004), persists into adulthood. In addition, also in agreement with Dąbrowska (2018, 2019), we provide further evidence that collocational knowledge in adult L1 speakers relates to both grammatical knowledge and lexical knowledge. These results, taken together, are fully compatible with the essential premise of usage-based models of language acquisition that all linguistic knowledge is represented in the same format, as pairings between forms and their meaning, and therefore depend on the same learning mechanisms and rely on the same memory systems (Tomasello, 2000; Langacker, 2009; Bybee, 2010). In contrast, our results are more difficult to accommodate in modular models (e.g., Chomsky, 1965; Pinker, 1994, 1999; Ullman et al., 1997; DeKeyser et al., 2010), which claim that grammar and vocabulary constitute different cognitive modules and are acquired using fundamentally different learning mechanisms.

## Conclusion

In sum, the present study shows that (explicit) phonological short-term memory and explicit memory for cross-modal associations, but not implicit memory for sequences, predict L1 linguistic knowledge in adults. This is the case for vocabulary, collocations, and most importantly, grammar. These results challenge the widely held assumption that L1 grammar learning depends almost entirely on the implicit memory system (Ellis, 1996; Ullman, 2001; DeKeyser et al., 2010). In addition, as argued above, our results are potentially problematic for modular theories of language acquisition because of the similarity of the relationships found between explicit memory predictors and language scores for grammatical, lexical and collocational knowledge, and the very strong relationships between scores in the three language tasks. Both of these findings align more closely with the view of language acquisition put forward by usage-based models.

## Data Availability Statement

All datasets presented in this study are included in the article/Supplementary Material.

## Ethics Statement

The studies involving human participants were reviewed and approved by Ethik-Kommission der Medizinischen Fakultät der FAU. Written informed consent for participation was not required for this study in accordance with the national legislation and the institutional requirements.

## Author Contributions

ML and ED conceptualized the study, ML implemented the experimental tasks and processed and analyzed the data. ML and ED drafted and revised the manuscript. Both authors contributed to the article and approved the submitted version.

## Conflict of Interest

The authors declare that the research was conducted in the absence of any commercial or financial relationships that could be construed as a potential conflict of interest.

## Footnotes

1 The terms *declarative/explicit* and *procedural/implicit* are not, strictly speaking, synonymous: the distinction between declarative and procedural relates to the brain systems, while the distinction between explicit and implicit is concerned with awareness. In the present paper, we only use the terms *declarative* and *procedural* in the introductory section, where we discuss the neurological underpinnings of memory. In the rest of the paper, we use the terms *explicit* and *implicit.*

2 Scores for the grammar task were calculated for all trials as well as only for trials with response times shorter than 5000 ms. This was done in order to ensure that individual scores were not simply a reflection of reading speed (i.e., lower scores caused by not being able to read the sentence in the 5000 ms window). Individual scores were almost identical for the two datasets, mainly because RT were in their vast majority shorter than 5000 ms (mean RT = 2717 ms). Because of this, we decided to use the dataset containing all trials for statistical analyses.

3 This analysis was also conducted on accuracy scores corrected for guessing following the formula outlined in Dąbrowska (2018). As the main results of interest did not differ from those of the analysis with raw proportions, only the latter are reported for the sake of easier interpretation of effects and regression coefficients.

4 The traditional measure of learning in the SRT task (Linck et al., 2013) was not included in the regression analysis in order to minimize multicollinearity. This decision was motivated by the fact that SRT slope appeared to be a more sensitive predictor of linguistic ability in the correlational analyses than the traditional subtraction measure.

5 As women have been reported to outperform men in tasks involving declarative memory as well as in language tasks (see Ullman et al., 2007 for a review) we examined whether sex differences in performance on the language and explicit memory tasks were apparent in the present study for the HAA group, where there were enough participants of each sex to make comparisons. However, no substantial differences were found in the means for women and men for any of the three language tasks nor for the Digit Span and LLAMA-B task.

6 The language tasks in this study presented stimuli in written form. Hence, participants’ reading abilities may have had an influence on their performance. This is especially the case for the grammar task, where participants were prompted to make grammaticality judgments quickly and reading difficulties could have possibly led to errors for participants in the LAA group. However, recent research in our lab suggests that grammaticality judgments by LAA speakers are not strongly affected by the modality of presentation (written vs. oral). Becker and Dąbrowska (2020) administered a grammaticality judgment task to a group of LAA speakers very similar to our participants and found no effects of modality for this group: in other words, the participants were equally good at judging sentences that were presented in writing and auditorily presented sentences. For this reason, we consider that the role that differences in reading abilities may have played in our results is a limited one.

7 A reviewer pointed out that the group differences observed in the grammar task could be partly due to the fact that the LAA group was likely to include more participants who spoke non-standard varieties of English. Although we cannot completely rule out this possibility, dialectal differences are unlikely to have had a significant effect on our results. As indicated in the Methods section, our stimuli for the grammaticality judgment task were taken from those used by Johnson and Newport (1989) and DeKeyser (2000). These studies were concerned with comparing the performance of native and non-native speakers, and hence the stimuli were designed to be as dialect-neutral as possible. However, after consulting with a dialectologist, we were made aware that four of the sentences which we had classified as ungrammatical are acceptable in some dialects of English. These items, however, did not discriminate between the HAA and LAA participants better than other items in the test.
