# Supplementary material for: Explicit but Not Implicit Memory Predicts Ultimate Attainment in the Native Language
Source: Front Psychol. 2020 Sep 25;11:569586. doi: 10.3389/fpsyg.2020.569586 (PMC7546274; doi:10.3389/fpsyg.2020.569586)
Supplement: Supplementary file 3 [file Data_Sheet_3.DOCX]

This code has been uploaded as a Word file because the submission system did not accept the original R script. Do not hesitate to contact the first author if you wish to obtain the original .R files.

library(readxl)

###First of all, set the path to the folder containing the dataframe

data <- read_excel("/YOUR PATH/mixed_models_L_M.xlsx")

library(lme4)

library(lmerTest)

###Contrast coding group (LAA vs. HAA)

data$groupC = ifelse(data$group=="low",-0.5,0.5)

data$group <- as.factor(data$group)

###Factor coding task, grammar as reference level

data$task <- as.factor(data$task)

data$task = relevel(data$task, ref=2)

###First run the most complex model (model summaries not provided in this script because of their length. Results of model comparisons are provided)

fm = lmer(acc ~ task + scale(srt) + scale(ll) + scale(ds) + scale(read) + groupC + task:groupC + task:scale(srt) + task:scale(ll) + task:scale(ds) + task:scale(read) + groupC:scale(srt) + groupC:scale(ll) + groupC:scale(ds) + groupC:scale(read) + task:groupC:scale(srt) + task:groupC:scale(ll) + task:groupC:scale(ds) + task:groupC:scale(read)+ (1|participant),data = data)

summary(fm)

###Remove three-way interaction: task x group x reading index

fm = lmer(acc ~ task + scale(srt) + scale(ll) + scale(ds) + scale(read) + groupC + task:groupC + task:scale(srt) + task:scale(ll) + task:scale(ds) + task:scale(read) + groupC:scale(srt) + groupC:scale(ll) + groupC:scale(ds) + groupC:scale(read) + task:groupC:scale(srt) + task:groupC:scale(ll) + task:groupC:scale(ds) + task:groupC:scale(read)+ (1|participant),data = data)

m1 = lmer(acc ~ task + scale(srt) + scale(ll) + scale(ds) + scale(read) + groupC + task:groupC + task:scale(srt) + task:scale(ll) + task:scale(ds) + task:scale(read) + groupC:scale(srt) + groupC:scale(ll) + groupC:scale(ds) + groupC:scale(read) + task:groupC:scale(srt) + task:groupC:scale(ll) + task:groupC:scale(ds) + (1|participant), data = data)

summary(m1)

###Log-likelihood test

anova(fm,m1)

# Df AIC BIC logLik deviance Chisq Chi Df Pr(>Chisq)

# m1 30 -328.37 -232.58 194.19 -388.37

# fm 32 -329.55 -227.38 196.78 -393.55 5.1786 2 0.07507 .

###Model with interaction not significantly better fitting than model without it: three-way interaction removed

### Remove three-way interaction: task x group x llamaB

m1 = lmer(acc ~ task + scale(srt) + scale(ll) + scale(ds) + scale(read) + groupC + task:groupC + task:scale(srt) + task:scale(ll) + task:scale(ds) + task:scale(read) + groupC:scale(srt) + groupC:scale(ll) + groupC:scale(ds) + groupC:scale(read) + task:groupC:scale(srt) + task:groupC:scale(ll) + task:groupC:scale(ds) + (1|participant), data = data)

m2 = lmer(acc ~ task + scale(srt) + scale(ll) + scale(ds) + scale(read) + groupC + task:groupC + task:scale(srt) + task:scale(ll) + task:scale(ds) + task:scale(read) + groupC:scale(srt) + groupC:scale(ll) + groupC:scale(ds) + groupC:scale(read) + task:groupC:scale(srt) + task:groupC:scale(ds) + (1|participant), data = data)

summary(m2)

anova(m1,m2)

# Df AIC BIC logLik deviance Chisq Chi Df Pr(>Chisq)

# m2 28 -331.21 -241.81 193.61 -387.21

# m1 30 -328.37 -232.58 194.19 -388.37 1.1587 2 0.5603

###Model with interaction not significantly better fitting than model without it: three-way interaction removed

### Remove three-way interaction: task x group x digit span.

m2 = lmer(acc ~ task + scale(srt) + scale(ll) + scale(ds) + scale(read) + groupC + task:groupC + task:scale(srt) + task:scale(ll) + task:scale(ds) + task:scale(read) + groupC:scale(srt) + groupC:scale(ll) + groupC:scale(ds) + groupC:scale(read) + task:groupC:scale(srt) + task:groupC:scale(ds) + (1|participant), data = data)

m3 = lmer(acc ~ task + scale(srt) + scale(ll) + scale(ds) + scale(read) + groupC + task:groupC + task:scale(srt) + task:scale(ll) + task:scale(ds) + task:scale(read) + groupC:scale(srt) + groupC:scale(ll) + groupC:scale(ds) + groupC:scale(read) + task:groupC:scale(srt) + (1|participant), data = data)

summary(m3)

anova(m2,m3)

# Df AIC BIC logLik deviance Chisq Chi Df Pr(>Chisq)

# m3 26 -334.64 -251.62 193.32 -386.64

# m2 28 -331.21 -241.81 193.61 -387.21 0.5757 2 0.7499

###Model with interaction not significantly better fitting than model without it: three-way interaction removed

### Remove three-way interaction: task x group x SRT.

m3 = lmer(acc ~ task + scale(srt) + scale(ll) + scale(ds) + scale(read) + groupC + task:groupC + task:scale(srt) + task:scale(ll) + task:scale(ds) + task:scale(read) + groupC:scale(srt) + groupC:scale(ll) + groupC:scale(ds) + groupC:scale(read) + task:groupC:scale(srt) + (1|participant), data = data)

m4 = lmer(acc ~ task + scale(srt) + scale(ll) + scale(ds) + scale(read) + groupC + task:groupC + task:scale(srt) + task:scale(ll) + task:scale(ds) + task:scale(read) + groupC:scale(srt) + groupC:scale(ll) + groupC:scale(ds) + groupC:scale(read) + (1|participant), data = data)

summary(m4)

anova(m3,m4)

# Df AIC BIC logLik deviance Chisq Chi Df Pr(>Chisq)

# m4 24 -338.20 -261.57 193.10 -386.20

# m3 26 -334.64 -251.62 193.32 -386.64 0.4351 2 0.8045

###Model with interaction not significantly better fitting than model without it: three-way interaction removed

### Remove interaction: group x reading index.

m4 = lmer(acc ~ task + scale(srt) + scale(ll) + scale(ds) + scale(read) + groupC + task:groupC + task:scale(srt) + task:scale(ll) + task:scale(ds) + task:scale(read) + groupC:scale(srt) + groupC:scale(ll) + groupC:scale(ds) + groupC:scale(read) + (1|participant), data = data)

m5 = lmer(acc ~ task + scale(srt) + scale(ll) + scale(ds) + scale(read) + groupC + task:groupC + task:scale(srt) + task:scale(ll) + task:scale(ds) + task:scale(read) + groupC:scale(srt) + groupC:scale(ll) + groupC:scale(ds) + (1|participant), data = data)

summary(m5)

anova(m4,m5)

# Df AIC BIC logLik deviance Chisq Chi Df Pr(>Chisq)

# m5 23 -340.1 -266.66 193.05 -386.1

# m4 24 -338.2 -261.57 193.10 -386.2 0.1031 1 0.7481

###Model with interaction not significantly better fitting than model without it: interaction removed

### Remove interaction: group x digit span

m5 = lmer(acc ~ task + scale(srt) + scale(ll) + scale(ds) + scale(read) + groupC + task:groupC + task:scale(srt) + task:scale(ll) + task:scale(ds) + task:scale(read) + groupC:scale(srt) + groupC:scale(ll) + groupC:scale(ds) + (1|participant), data = data)

m6 = lmer(acc ~ task + scale(srt) + scale(ll) + scale(ds) + scale(read) + groupC + task:groupC + task:scale(srt) + task:scale(ll) + task:scale(ds) + task:scale(read) + groupC:scale(srt) + groupC:scale(ll) + (1|participant), data = data)

summary(m6)

anova(m5,m6)

# Df AIC BIC logLik deviance Chisq Chi Df Pr(>Chisq)

# m6 22 -340.85 -270.60 192.42 -384.85

# m5 23 -340.10 -266.66 193.05 -386.10 1.2536 1 0.2629

###Model with interaction not significantly better fitting than model without it: interaction removed

### Remove interaction: group x llamaB

m6 = lmer(acc ~ task + scale(srt) + scale(ll) + scale(ds) + scale(read) + groupC + task:groupC + task:scale(srt) + task:scale(ll) + task:scale(ds) + task:scale(read) + groupC:scale(srt) + groupC:scale(ll) + (1|participant), data = data)

m7 = lmer(acc ~ task + scale(srt) + scale(ll) + scale(ds) + scale(read) + groupC + task:groupC + task:scale(srt) + task:scale(ll) + task:scale(ds) + task:scale(read) + groupC:scale(srt) + (1|participant), data = data)

summary(m7)

anova(m6,m7)

# Df AIC BIC logLik deviance Chisq Chi Df Pr(>Chisq)

# m7 21 -335.86 -268.81 188.93 -377.86

# m6 22 -340.85 -270.60 192.42 -384.85 6.9858 1 0.008216 **

###Model with interaction fits the data significantly better than model without it: interaction kept in further comparisons

### Remove interaction: group x SRT

m6 = lmer(acc ~ task + scale(srt) + scale(ll) + scale(ds) + scale(read) + groupC + task:groupC + task:scale(srt) + task:scale(ll) + task:scale(ds) + task:scale(read) + groupC:scale(srt) + groupC:scale(ll) + (1|participant), data = data)

m8 = lmer(acc ~ task + scale(srt) + scale(ll) + scale(ds) + scale(read) + groupC + task:groupC + task:scale(srt) + task:scale(ll) + task:scale(ds) + task:scale(read) + groupC:scale(ll) + (1|participant), data = data)

summary(m8)

anova(m6,m8)

# Df AIC BIC logLik deviance Chisq Chi Df Pr(>Chisq)

# m8 21 -342.45 -275.4 192.22 -384.45

# m6 22 -340.85 -270.6 192.42 -384.85 0.3984 1 0.5279

###Model with interaction not significantly better fitting than model without it: interaction removed

### Remove interaction: task x reading index

m8 = lmer(acc ~ task + scale(srt) + scale(ll) + scale(ds) + scale(read) + groupC + task:groupC + task:scale(srt) + task:scale(ll) + task:scale(ds) + task:scale(read) + groupC:scale(ll) + (1|participant), data = data)

m9 = lmer(acc ~ task + scale(srt) + scale(ll) + scale(ds) + scale(read) + groupC + task:groupC + task:scale(srt) + task:scale(ll) + task:scale(ds) + groupC:scale(ll) + (1|participant), data = data)

summary(m9)

anova(m8,m9)

# Df AIC BIC logLik deviance Chisq Chi Df Pr(>Chisq)

# m9 19 -343.35 -282.68 190.67 -381.35

# m8 21 -342.45 -275.40 192.22 -384.45 3.0997 2 0.2123

###Model with interaction not significantly better fitting than model without it: interaction removed

### Remove interaction: task x digit span

m9 = lmer(acc ~ task + scale(srt) + scale(ll) + scale(ds) + scale(read) + groupC + task:groupC + task:scale(srt) + task:scale(ll) + task:scale(ds) + groupC:scale(ll) + (1|participant), data = data)

m10 = lmer(acc ~ task + scale(srt) + scale(ll) + scale(ds) + scale(read) + groupC + task:groupC + task:scale(srt) + task:scale(ll) + groupC:scale(ll) + (1|participant), data = data)

summary(m10)

anova(m9,m10)

# Df AIC BIC logLik deviance Chisq Chi Df Pr(>Chisq)

# m10 17 -345.20 -290.92 189.60 -379.20

# m9 19 -343.35 -282.68 190.67 -381.35 2.148 2 0.3416

###Model with interaction not significantly better fitting than model without it: interaction removed

### Remove interaction: task x llamaB

m10 = lmer(acc ~ task + scale(srt) + scale(ll) + scale(ds) + scale(read) + groupC + task:groupC + task:scale(srt) + task:scale(ll) + groupC:scale(ll) + (1|participant), data = data)

m11 = lmer(acc ~ task + scale(srt) + scale(ll) + scale(ds) + scale(read) + groupC + task:groupC + task:scale(srt) + groupC:scale(ll) + (1|participant), data = data)

summary(m11)

anova(m10,m11)

# Df AIC BIC logLik deviance Chisq Chi Df Pr(>Chisq)

# m11 15 -348.83 -300.94 189.42 -378.83

# m10 17 -345.20 -290.92 189.60 -379.20 0.3668 2 0.8324

###Model with interaction not significantly better fitting than model without it: interaction removed

### Remove interaction: task x SRT

m11 = lmer(acc ~ task + scale(srt) + scale(ll) + scale(ds) + scale(read) + groupC + task:groupC + task:scale(srt) + groupC:scale(ll) + (1|participant), data = data)

m12 = lmer(acc ~ task + scale(srt) + scale(ll) + scale(ds) + scale(read) + groupC + task:groupC + groupC:scale(ll) + (1|participant), data = data)

summary(m12)

anova(m11,m12)

# Df AIC BIC logLik deviance Chisq Chi Df Pr(>Chisq)

# m12 13 -350.37 -308.86 188.18 -376.37

# m11 15 -348.83 -300.94 189.42 -378.83 2.4678 2 0.2911

###Model with interaction not significantly better fitting than model without it: interaction removed

### Remove interaction: task x group.

m12 = lmer(acc ~ task + scale(srt) + scale(ll) + scale(ds) + scale(read) + groupC + task:groupC + groupC:scale(ll) + (1|participant), data = data)

m13 = lmer(acc ~ task + scale(srt) + scale(ll) + scale(ds) + scale(read) + groupC + groupC:scale(ll) + (1|participant), data = data)

summary(m13)

anova(m12,m13)

# Df AIC BIC logLik deviance Chisq Chi Df Pr(>Chisq)

# m13 11 -344.72 -309.60 183.36 -366.72

# m12 13 -350.37 -308.86 188.18 -376.37 9.6418 2 0.00806 **

###Model with interaction fits the data significantly better than model without it: interaction kept in further comparisons

### Remove group

m12 = lmer(acc ~ task + scale(srt) + scale(ll) + scale(ds) + scale(read) + groupC + groupC:scale(ll) + (1|participant), data = data)

m14 = lmer(acc ~ task + scale(srt) + scale(ll) + scale(ds) + scale(read) + groupC:scale(ll) + (1|participant), data = data)

summary(m14)

anova(m12,m14)

# Df AIC BIC logLik deviance Chisq Chi Df Pr(>Chisq)

# m14 10 -321.66 -289.73 170.83 -341.66

# m12 11 -344.72 -309.60 183.36 -366.72 25.064 1 5.547e-07 ***

###Model with group fits the data significantly better than model without it: group kept in further comparisons

### Remove reading index

m12 = lmer(acc ~ task + scale(srt) + scale(ll) + scale(ds) + scale(read) + groupC + task:groupC + groupC:scale(ll) + (1|participant), data = data)

m15 = lmer(acc ~ task + scale(srt) + scale(ll) + scale(ds) + groupC + task:groupC + groupC:scale(ll) + (1|participant), data = data)

summary(m15)

anova(m12,m15)

# Df AIC BIC logLik deviance Chisq Chi Df Pr(>Chisq)

# m15 12 -351.83 -313.51 187.91 -375.83

# m12 13 -350.37 -308.86 188.18 -376.37 0.5398 1 0.4625

###Model with reading index not significantly better fitting than model without it: reading index removed

### Remove digit span

m15 = lmer(acc ~ task + scale(srt) + scale(ll) + scale(ds) + groupC + task:groupC + groupC:scale(ll) + (1|participant), data = data)

m16 = lmer(acc ~ task + scale(srt) + scale(ll) + groupC + task:groupC + groupC:scale(ll) + (1|participant), data = data)

summary(m16)

anova(m15,m16)

# Df AIC BIC logLik deviance Chisq Chi Df Pr(>Chisq)

# m16 11 -341.40 -306.27 181.70 -363.40

# m15 12 -351.83 -313.51 187.91 -375.83 12.431 1 0.0004222 ***

###Model with digit span fits the data significantly better than model without it: digit span kept in further comparisons

### Remove llamaB

m15 = lmer(acc ~ task + scale(srt) + scale(ll) + scale(ds) + groupC + task:groupC + groupC:scale(ll) + (1|participant), data = data)

m17 = lmer(acc ~ task + scale(srt) + scale(ds) + groupC + task:groupC + groupC:scale(ll) + (1|participant), data = data)

summary(m17)

anova(m15,m17)

# Df AIC BIC logLik deviance Chisq Chi Df Pr(>Chisq)

# m17 11 -341.85 -306.73 181.93 -363.85

# m15 12 -351.83 -313.51 187.91 -375.83 11.976 1 0.0005389 ***

###Model with llamaB fits the data significantly better than model without it: llamaB kept in further comparisons

### Remove SRT

m15 = lmer(acc ~ task + scale(srt) + scale(ll) + scale(ds) + groupC + task:groupC + groupC:scale(ll) + (1|participant), data = data)

m18 = lmer(acc ~ task + scale(ll) + scale(ds) + groupC + task:groupC + groupC:scale(ll) + (1|participant), data = data)

summary(m18)

anova(m15,m18)

# Df AIC BIC logLik deviance Chisq Chi Df Pr(>Chisq)

# m18 11 -353.66 -318.54 187.83 -375.66

# m15 12 -351.83 -313.51 187.91 -375.83 0.1686 1 0.6814

###Model with SRT not significantly better fitting than model without it: SRT removed

### Remove task

m18 = lmer(acc ~ task + scale(ll) + scale(ds) + groupC + task:groupC + groupC:scale(ll) + (1|participant), data = data)

m19 = lmer(acc ~ scale(ll) + scale(ds) + groupC + task:groupC + groupC:scale(ll) + (1|participant), data = data)

summary(m19)

anova(m18,m19)

# Df AIC BIC logLik deviance Chisq Chi Df Pr(>Chisq)

# m19 9 -209.64 -180.91 113.82 -227.64

# m18 11 -353.66 -318.54 187.83 -375.66 148.01 2 < 2.2e-16 ***

###Model with task fits the data significantly better than model without it: task kept in final model

#model 18 == Final model

finalM = lmer(acc ~ task + scale(ll) + scale(ds) + groupC + task:groupC + groupC:scale(ll) + (1|participant), data = data)

summary(finalM)

# Linear mixed model fit by REML. t-tests use Satterthwaite's method ['lmerModLmerTest']

# Formula: acc ~ task + scale(ll) + scale(ds) + groupC + task:groupC + groupC:scale(ll) + (1 | participant)

# Data: data

#

# REML criterion at convergence: -316.3

#

# Scaled residuals:

# Min 1Q Median 3Q Max

# -3.4045 -0.5044 -0.0050 0.5397 3.0846

#

# Random effects:

# Groups Name Variance Std.Dev.

# participant (Intercept) 0.001345 0.03668

# Residual 0.006513 0.08070

# Number of obs: 180, groups: participant, 60

#

# Fixed effects:

# Estimate Std. Error df t value Pr(>|t|)

# (Intercept) 0.85452 0.01472 120.47338 58.064 < 2e-16 ***

# taskcolloc -0.24604 0.01563 115.99999 -15.744 < 2e-16 ***

# taskvocab -0.14979 0.01563 115.99999 -9.585 2.23e-16 ***

# scale(ll) 0.03866 0.01114 55.00001 3.472 0.001014 **

# scale(ds) 0.03361 0.00905 55.00001 3.714 0.000478 ***

# groupC 0.09180 0.02963 119.29472 3.098 0.002429 **

# taskcolloc:groupC 0.09708 0.03125 115.99999 3.106 0.002383 **

# taskvocab:groupC 0.04208 0.03125 115.99999 1.346 0.180785

# scale(ll):groupC -0.06069 0.02184 55.00001 -2.779 0.007442 **

# ---

# Signif. codes: 0 ‘***’ 0.001 ‘**’ 0.01 ‘*’ 0.05 ‘.’ 0.1 ‘ ’ 1

#

# Correlation of Fixed Effects:

# (Intr) tskcll tskvcb scl(l) scl(d) groupC tskc:C tskv:C

# taskcolloc -0.531

# taskvocab -0.531 0.500

# scale(ll) 0.347 0.000 0.000

# scale(ds) -0.047 0.000 0.000 -0.280

# groupC -0.472 0.176 0.176 -0.484 -0.125

# tskcllc:grC 0.177 -0.333 -0.167 0.000 0.000 -0.527

# tskvcb:grpC 0.177 -0.167 -0.333 0.000 0.000 -0.527 0.500

# scl(ll):grC -0.542 0.000 0.000 -0.422 0.203 0.313 0.000 0.000

###Subset the dataset by task so that each resulting dataset has 60 rows (one per participant) and only includes scores from one of the language tasks.

grammar = subset(data, data$task == "grammar")

vocab = subset(data, data$task == "vocab")

colloc = subset(data, data$task == "colloc")

###Run one linear regression model on each dataset (each language task). The first one is grammar.

mgramSh = lm(acc ~ scale(ll) + scale(ds) + groupC + groupC:scale(ll), data = grammar)

summary(mgramSh)

#

# Call:

# lm(formula = acc ~ scale(ll) + scale(ds) + groupC + groupC:scale(ll),

# data = grammar)

#

# Residuals:

# Min 1Q Median 3Q Max

# -0.201671 -0.032001 0.003935 0.029098 0.199349

#

# Coefficients:

# Estimate Std. Error t value Pr(>|t|)

# (Intercept) 0.855059 0.011825 72.307 < 2e-16 ***

# scale(ll) 0.035247 0.011389 3.095 0.003094 **

# scale(ds) 0.030684 0.009256 3.315 0.001626 **

# groupC 0.098378 0.023906 4.115 0.000131 ***

# scale(ll):groupC -0.066605 0.022333 -2.982 0.004258 **

# ---

# Signif. codes: 0 ‘***’ 0.001 ‘**’ 0.01 ‘*’ 0.05 ‘.’ 0.1 ‘ ’ 1

#

# Residual standard error: 0.06031 on 55 degrees of freedom

# Multiple R-squared: 0.7499, Adjusted R-squared: 0.7318

# F-statistic: 41.24 on 4 and 55 DF, p-value: 6.04e-16

###Linear regression model on vocabulary data.

mvocSh = lm(acc ~ scale(ll) + scale(ds) + groupC + groupC:scale(ll), data = vocab)

summary(mvocSh)

#

# Call:

# lm(formula = acc ~ scale(ll) + scale(ds) + groupC + groupC:scale(ll),

# data = vocab)

#

# Residuals:

# Min 1Q Median 3Q Max

# -0.193131 -0.063461 0.004227 0.065018 0.253967

#

# Coefficients:

# Estimate Std. Error t value Pr(>|t|)

# (Intercept) 0.70709 0.01785 39.620 < 2e-16 ***

# scale(ll) 0.04402 0.01719 2.561 0.013209 *

# scale(ds) 0.02358 0.01397 1.688 0.097040 .

# groupC 0.13508 0.03608 3.744 0.000435 ***

# scale(ll):groupC -0.06975 0.03371 -2.069 0.043211 *

# ---

# Signif. codes: 0 ‘***’ 0.001 ‘**’ 0.01 ‘*’ 0.05 ‘.’ 0.1 ‘ ’ 1

#

# Residual standard error: 0.09102 on 55 degrees of freedom

# Multiple R-squared: 0.6468, Adjusted R-squared: 0.6211

# F-statistic: 25.18 on 4 and 55 DF, p-value: 7.009e-12

#

###Linear regression model on collocations data.

mcollSh = lm(acc ~ scale(ll) + scale(ds) + groupC + groupC:scale(ll), data = colloc)

summary(mcollSh)

# Call:

# lm(formula = acc ~ scale(ll) + scale(ds) + groupC + groupC:scale(ll),

# data = colloc)

#

# Residuals:

# Min 1Q Median 3Q Max

# -0.305059 -0.055465 -0.008781 0.074227 0.221984

#

# Coefficients:

# Estimate Std. Error t value Pr(>|t|)

# (Intercept) 0.60558 0.02156 28.089 < 2e-16 ***

# scale(ll) 0.03737 0.02076 1.800 0.077421 .

# scale(ds) 0.04713 0.01688 2.793 0.007177 **

# groupC 0.18112 0.04358 4.156 0.000114 ***

# scale(ll):groupC -0.04673 0.04072 -1.148 0.256124

# ---

# Signif. codes: 0 ‘***’ 0.001 ‘**’ 0.01 ‘*’ 0.05 ‘.’ 0.1 ‘ ’ 1

#

# Residual standard error: 0.11 on 55 degrees of freedom

# Multiple R-squared: 0.6583, Adjusted R-squared: 0.6335

# F-statistic: 26.5 on 4 and 55 DF, p-value: 2.85e-12
