# Supplementary material for: Explicit but Not Implicit Memory Predicts Ultimate Attainment in the Native Language
Source: Front Psychol. 2020 Sep 25;11:569586. doi: 10.3389/fpsyg.2020.569586 (PMC7546274; doi:10.3389/fpsyg.2020.569586)
Supplement: Supplementary file 4 [file Data_Sheet_4.DOCX]

This code has been uploaded as a Word file because the submission system did not accept the original R script. Do not hesitate to contact the first author if you wish to obtain the original .R files.

library(readxl)

###First of all, set the path to the folder containing the dataframe

ind <- read_excel("YOUR PATH/individual_values_L_M.xlsx")

attach(ind)

####T-tests comparing performances by HAA and LAA participants for all individual measures collected

###Grammar

grammar <- t.test(gjt_eng ~ group, data = ind)

grammar

# Welch Two Sample t-test

#

# data: gjt_eng by group

# t = 7.1283, df = 20.66, p-value = 5.466e-07

# alternative hypothesis: true difference in means is not equal to 0

# 95 percent confidence interval:

# 0.1373088 0.2505860

# sample estimates:

# mean in group HAA mean in group LAA

# 0.9150000 0.7210526

###Vocabulary

vocab <- t.test(voc_eng ~ group, data = ind)

vocab

# Welch Two Sample t-test

#

# data: voc_eng by group

# t = 8.1512, df = 27.68, p-value = 7.769e-09

# alternative hypothesis: true difference in means is not equal to 0

# 95 percent confidence interval:

# 0.1817737 0.3038842

# sample estimates:

# mean in group HAA mean in group LAA

# 0.7862500 0.5434211

###Collocations

coll <- t.test(coll_eng ~ group, data = ind)

coll

# Welch Two Sample t-test

#

# data: coll_eng by group

# t = 8.393, df = 30.005, p-value = 2.284e-09

# alternative hypothesis: true difference in means is not equal to 0

# 95 percent confidence interval:

# 0.2253088 0.3702175

# sample estimates:

# mean in group HAA mean in group LAA

# 0.7175000 0.4197368

###Digit Span

ds <- t.test(digit_span ~ group, data = ind)

ds

# Welch Two Sample t-test

#

# data: digit_span by group

# t = 3.8323, df = 33.481, p-value = 0.0005313

# alternative hypothesis: true difference in means is not equal to 0

# 95 percent confidence interval:

# 0.07800796 0.25436145

# sample estimates:

# mean in group HAA mean in group LAA

# 0.5897436 0.4235589

###LLAMA

llama <- t.test(LLAMA ~ group, data = ind)

llama

# Welch Two Sample t-test

#

# data: LLAMA by group

# t = 6.2291, df = 39.878, p-value = 2.279e-07

# alternative hypothesis: true difference in means is not equal to 0

# 95 percent confidence interval:

# 0.2254515 0.4420485

# sample estimates:

# mean in group HAA mean in group LAA

# 0.60875 0.27500

###Serial Reaction Time, traditional measure

srtt <- t.test(SRT ~ group, data = ind)

srtt

#

# Welch Two Sample t-test

#

# data: SRT by group

# t = 0.93714, df = 21.9, p-value = 0.3589

# alternative hypothesis: true difference in means is not equal to 0

# 95 percent confidence interval:

# -23.56726 62.40685

# sample estimates:

# mean in group HAA mean in group LAA

# 22.417068 2.997272

###Serial Reaction Time, slope

srtSl <- t.test(srt_slope ~ group, data = ind)

srtSl

# Welch Two Sample t-test

#

# data: srt_slope by group

# t = -1.9223, df = 33.341, p-value = 0.06314

# alternative hypothesis: true difference in means is not equal to 0

# 95 percent confidence interval:

# -1.215504e-03 3.422866e-05

# sample estimates:

# mean in group HAA mean in group LAA

# -5.698449e-04 2.079297e-05

###Reading Index

read <- t.test(readMix ~ group, data = ind)

read

# Welch Two Sample t-test

#

# data: readMix by group

# t = 6.3331, df = 52.14, p-value = 5.655e-08

# alternative hypothesis: true difference in means is not equal to 0

# 95 percent confidence interval:

# 1.272406 2.452594

# sample estimates:

# mean in group high mean in group low

# 3.7375 1.8750

####Correlation matrix for all measures collected

library(Hmisc)

###Participant and Group columns removed from dataframe

drops <- c("participant","group")

ind <-ind[ , !(names(ind) %in% drops)]

###Get correlation matrix

mycor <- rcorr(as.matrix(ind), type="pearson")

mycor

# coll_eng gjt_eng voc_eng digit_span SRT LLAMA srt_slope readMix

# coll_eng 1.00 0.80 0.82 0.60 0.20 0.61 -0.28 0.35

# gjt_eng 0.80 1.00 0.70 0.64 0.12 0.65 -0.20 0.32

# voc_eng 0.82 0.70 1.00 0.56 0.18 0.63 -0.34 0.48

# digit_span 0.60 0.64 0.56 1.00 0.23 0.44 -0.31 0.17

# SRT 0.20 0.12 0.18 0.23 1.00 0.11 -0.56 0.20

# LLAMA 0.61 0.65 0.63 0.44 0.11 1.00 -0.13 0.29

# srt_slope -0.28 -0.20 -0.34 -0.31 -0.56 -0.13 1.00 -0.35

# readMix 0.35 0.32 0.48 0.17 0.20 0.29 -0.35 1.00

#

# n

# coll_eng gjt_eng voc_eng digit_span SRT LLAMA srt_slope readMix

# coll_eng 59 58 58 57 58 59 59 59

# gjt_eng 58 59 58 57 58 59 59 59

# voc_eng 58 58 59 57 58 59 59 59

# digit_span 57 57 57 58 57 58 58 58

# SRT 58 58 58 57 59 59 59 59

# LLAMA 59 59 59 58 59 60 60 60

# srt_slope 59 59 59 58 59 60 60 60

# readMix 59 59 59 58 59 60 60 60

#

# P

# coll_eng gjt_eng voc_eng digit_span SRT LLAMA srt_slope readMix

# coll_eng 0.0000 0.0000 0.0000 0.1396 0.0000 0.0343 0.0058

# gjt_eng 0.0000 0.0000 0.0000 0.3691 0.0000 0.1319 0.0124

# voc_eng 0.0000 0.0000 0.0000 0.1808 0.0000 0.0091 0.0001

# digit_span 0.0000 0.0000 0.0000 0.0886 0.0006 0.0164 0.1927

# SRT 0.1396 0.3691 0.1808 0.0886 0.4266 0.0000 0.1212

# LLAMA 0.0000 0.0000 0.0000 0.0006 0.4266 0.3304 0.0265

# srt_slope 0.0343 0.1319 0.0091 0.0164 0.0000 0.3304 0.0061

# readMix 0.0058 0.0124 0.0001 0.1927 0.1212 0.0265 0.0061

detach(ind)
