# Supplementary material for: Explicit but Not Implicit Memory Predicts Ultimate Attainment in the Native Language
Source: Front Psychol. 2020 Sep 25;11:569586. doi: 10.3389/fpsyg.2020.569586 (PMC7546274; doi:10.3389/fpsyg.2020.569586)
Supplement: Supplementary file 5 [file Table_1.docx]

Supplementary Materials

Table S1*. Coefficients and significance values for the linear regression model assessing the effects of Digit Span, LLAMA-B, Group and the interaction between LLAMA-B and Group on accuracy in the grammar task.*

| Predictor | *b* |  | | *t* |  | | *p* |
| --- | --- | --- | --- | --- | --- | --- | --- |
| Intercept | 0.90 | | 80.61 | | | < .001 | |
| Digit Span Score | 0.03 | | 3.32 | | | < .01 | |
| LLAMA-B Score | 0.04 | | 3.01 | | | < .01 | |
| Group | 0.10 | | 4.12 | | | < .001 | |
| LLAMA-B x Group | -0.07 | | -2.98 | | | < .01 | |
| Multiple R-squared: 0.75 | | | Adjusted R-squared: 0.73 | | | | |

Table S2*. Coefficients and significance values for the linear regression model assessing the effects of Digit Span, LLAMA-B, Group and the interaction between LLAMA-B and Group on accuracy in the vocabulary task.*

| Predictor | *b* |  | | *t* |  | | *p* |
| --- | --- | --- | --- | --- | --- | --- | --- |
| Intercept | 0.71 | | 39.62 | | | < .001 | |
| Digit Span Score | 0.02 | | 1.69 | | | .097 | |
| LLAMA-B Score | 0.04 | | 2.56 | | | < .05 | |
| Group | 0.14 | | 3.74 | | | < .001 | |
| LLAMA-B x Group | -0.07 | | -2.07 | | | < .05 | |
| Multiple R-squared: 0.65 | | | Adjusted R-squared: 0.62 | | | | |

Table S3*. Coefficients and significance values for the linear regression model assessing the effects of Digit Span, LLAMA-B, Group and the interaction between LLAMA-B and Group on accuracy in the collocations task.*

| Predictor | *b* |  | | *t* |  | | *p* |
| --- | --- | --- | --- | --- | --- | --- | --- |
| Intercept | 0.70 | | 34.04 | | | < .001 | |
| Digit Span Score | 0.05 | | 2.79 | | | <.01 | |
| LLAMA-B Score | 0.04 | | 1.80 | | | .077 | |
| Group | 0.18 | | 4.16 | | | < .001 | |
| LLAMA-B x Group | -0.05 | | -1.15 | | | .25 | |
| Multiple R-squared: 0.66 | | | Adjusted R-squared: 0.63 | | | | |

Items presented in the grammaticality judgment task by condition (grammatical / ungrammatical).

Grammatical

Sandy filled a jar with cookies last night.

Last night the books fell off the shelves.

Yesterday the teacher sent Allison to the principal.

Many houses were destroyed by the flood last week.

Two mice ran into the house this morning.

Our neighbor bought new furniture last week.

Mrs. Sampson cleans her house every Wednesday.

Mary will go to Europe next year.

The Johnsons may be moving to Chicago this fall.

Janet is wearing the dress I gave her.

The children are playing in the garden till dark these days.

Mrs. Johnson went to the library yesterday.

After a life like that he will go straight to hell.

Mary looked at the flowers but didn’t buy them.

Peter did not have any money on him.

The drunk slept off his hangover in the guest room.

The man looked the new cars over yesterday.

The little boys laughed at the clown.

I hope you will go to the store now.

Has the King been served his dinner?

Can the little girl ride a bicycle?

Does John know the answer to that question?

Where did Arnie hunt last year?

Where is Ted working this summer?

What do they sell at the corner store?

The boy caught the ball.

The boy feeds the rabbits carrots.

The dog bites.

The children play with the dog.

The student eats his meals quickly.

Ungrammatical

Last night the old lady die in her sleep.

Janie sleeped with her teddy bear last night.

A bat flewed into our attic last night.

Three boy played on the swings in the park.

A shoe salesman sees many foots throughout the day.

I need to get some informations about the train schedule.

John’s dog always wait for him at the corner.

John can plays the piano very well.

Our new neighbor should turns his radio down a bit.

The boy has been lie to his father.

Tom working in his office right now.

Tom is reading book in the bathtub.

The beauty is something that lasts forever.

Peter made out the check but didn’t sign.

The girl cut himself on a piece of glass.

The man climbed the ladder up carefully.

Kevin called Nancy for a date up.

George says much too softly.

The girls enjoy to watch TV.

Will be Harry blamed for the accident?

Will wear Harry his new shirt to the party?

Swam Janet in the race yesterday?

Did Bobbie stayed at home last night?

What Martha is bringing to the party?

Who you meet at the park every day?

The dinner the man burned.

The woman the policeman asked a question.

Drinks the man.

The students to the movies went.

Kevin rides usually his bicycle to work.
